# In Vivo Oncolytic Virotherapy in Murine Models of Hepatocellular Carcinoma: A Systematic Review

**DOI:** 10.3390/vaccines10091541

**Published:** 2022-09-16

**Authors:** Muhammad Joan Ailia, So Young Yoo

**Affiliations:** BIO-IT Foundry Technology Institute, Pusan National University, Busan 46241, Korea

**Keywords:** hepatocellular carcinoma, in vivo, murine models, oncolytic virotherapy

## Abstract

Hepatocellular carcinoma (HCC) is the third leading cause of cancer-related mortality worldwide. Current therapies often provide marginal survival benefits at the expense of undesirable side effects. Oncolytic viruses represent a novel strategy for the treatment of HCC due to their inherent ability to cause direct tumor cell lysis while sparing normal tissue and their capacity to stimulate potent immune responses directed against uninfected tumor cells and distant metastases. Oncolytic virotherapy (OVT) is a promising cancer treatment, but before it can become a standard option in practice, several challenges—systemic viral delivery optimization/enhancement, inter-tumoral virus dispersion, anti-cancer immunity cross-priming, and lack of artificial model systems—need to be addressed. Addressing these will require an in vivo model that accurately mimics the tumor microenvironment and allows the scientific community to design a more precise and accurate OVT. Due to their close physiologic resemblance to humans, murine cancer models are the likely preferred candidates. To provide an accurate assessment of the current state of in vivo OVT in HCC, we have reviewed a comprehensively searched body of work using murine in vivo HCC models for OVT.

## 1. Introduction

Hepatocellular carcinoma (HCC) is the third most fatal cancer globally, and existing therapies frequently deliver only minor survival gains at the expense of unfavorable side effects [1,2]. Immune-based cancer therapy has seen significant progress in recent years, promising safe, systemic, and long-term tumor responses. However, the challenges posed by the complex and immune-suppressive microenvironment of the liver make HCC a particularly difficult treatment target [3,4].

Oncolytic viruses (OVs) offer a novel strategy for the treatment of HCC. The OVs have an inherent ability to cause direct tumor cell lysis while sparing normal tissue, as well as their capacity to stimulate potent immune responses directed against uninfected tumor cells and distant metastases, resulting in an elegant multimodal therapy [4,5]. Thus, OVs provide a perfect platform for debulking the tumor, inducing immunological tolerance, and initiating potent anti-cancer immune-mediated effects, and their effects can be further enhanced with combination therapy [6]. The first clearance of an oncolytic virus by the United States Food and Drug Administration in 2015, virustalimogene laherparepvec (T-VEC), also known as OncoVEXGM-CSF (marketed by Amgen Inc. as Imlygic^®^), sparked a boom of interest in the OV research and development [7]. With the increased interest in and acceptance of OV therapies as potentially transformative cancer immunotherapies, developing enhanced, next-generation OVs has become a priority [8]. There have been reports of OV platforms under development for colorectal, lung, liver, bile duct, pancreatic, and gastric cancers. The field is gripped by a debate over which viral platforms are the most promising [6,9,10].

The murine model is one of the closely physiologically resembling animal models available for cancer studies [11]. Several murine models are very useful for research, including syngeneic murine models, genetically engineered mice models (GEMMs), human knock-in (KI) mice, human xenografts, patient-derived xenografts (PDX), Immuno-avatars, hematolymphoid humanized mice, and immunological PDX mice [12]. Among these, cell line-based xenograft models are the most cost-effective and commonly employed. Even though xenotransplantation of cell lines is simple and inexpensive, it should be performed in immunocompromised mice to avoid immune rejection, which makes them less ideal for studying due to the involvement of the immune system in tumor formation and therapy response [13]. In this instance, GEMs or human KI mice can be the model contributing to the improvement of oncolytic virotherapy (OVT).

To help navigate this field of competing approaches, we have compiled the first comprehensive review of in vivo HCC murine models currently used for OVT. We have identified five important groups of OVs and evaluated the associated approaches to hepatic cancer therapy.

## 2. Materials and Methods

### 2.1. Search Strategy

This study was conducted in accordance with the Preferred Reporting Items for Systematic Reviews and Meta-Analyses (PRISMA) and registered in PROSPERO (CRD42022325107). Four major electronic databases, Medline, EMBASE, Web of Science, and Cochrane Library, were searched for relevant English-language papers published through April 2022. The major keyword searched were “Oncolytic virotherapy”, “Oncolytic virus therapy", “Hepatocellular cancer”, “Liver cancer”, “Hepatic malignancy”, Hepatic neoplasm” and “Liver neoplasm”. The electronic search was followed by a manual search of the references by cross-referencing key papers. The database of the retrieved material was managed using EndNote X20 (Thomson Reuters, New York, NY, USA).

### 2.2. Criteria for Inclusion and Exclusion

The studies were included if they met all of the following 6 eligibility criteria: they (1) addressed OVT in HCC; (2) included sufficient information about the virus therapy; (3) used a novel or unique method or technique for virotherapy; (4) demonstrated an association between oncolytic therapies, cancer progression, tumor microenvironment (TME), and immune checkpoints; (5) were written in English; (6) obtained appropriate ethical approval for animal experiments.

The studies were excluded if they met any of the following 3 exclusion criteria: they (1) were duplicate studies, reviews, case reports, letters, or conference proceedings; (2) found no association between HCC and OV; or (3) lacked sufficient data on OV.

### 2.3. Data Extraction and Assessment of Study Quality

The data were extracted independently by the two authors (MJA and SYY). They used consensus to resolve their disagreements during the process. Metadata extracted from all studies included the following ten records: (1) the author/year, (2) country, (3) publication year, (4) agent/virus name, (5) virus type, (6) genome size, (7) specimen used, (8) laboratory animals, (9) administration route, and (10) outcome.

## 3. Results

### 3.1. Eligible Studies

**Figure 1:**
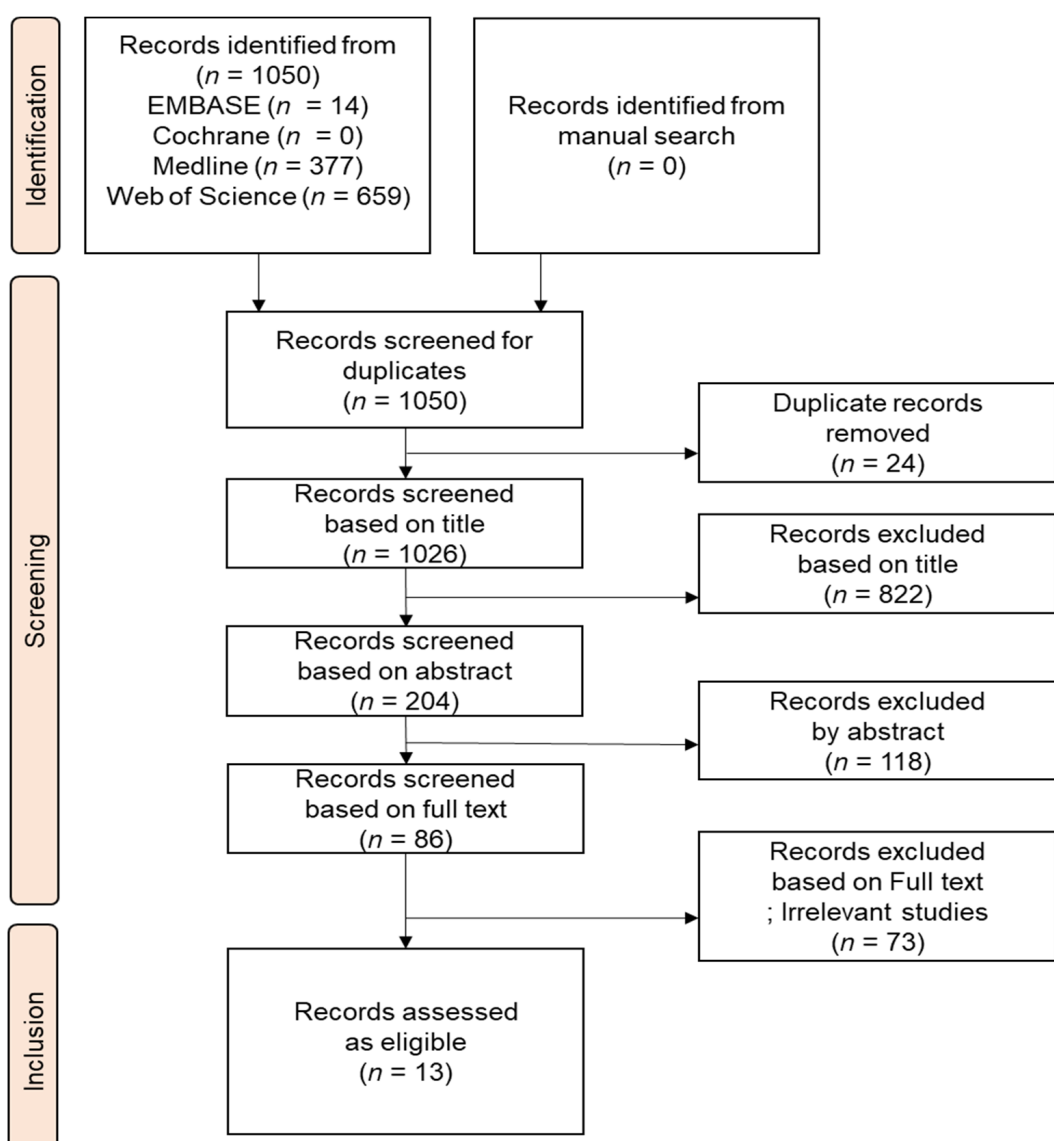
PRISMA flowchart of the search and selection procedure used to identify the studies reviewed here.

The retrieval approach described in Methods (Section 2) initially yielded 1050 studies, of which 24 were excluded based on duplicates, and 940 by their titles and abstracts. After reviewing the full texts of the remaining articles, a further 73 were excluded owing to missing, irrelevant, irretrievable, or duplicate data, leaving 13 studies for inclusion. The selection of studies was carried out in accordance with the PRISMA flowchart (Figure 1).

### 3.2. Characteristics of the Studies Included

**Figure 2:**
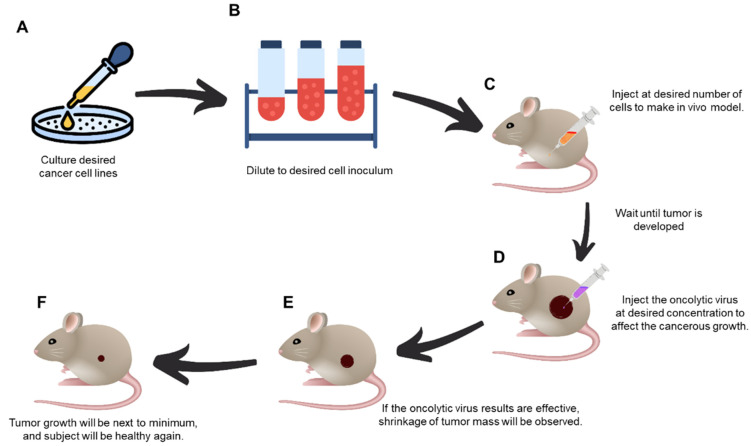
Schematics of the major procedural steps (**A**–**F**) of in vivo experimental oncolytic virotherapy. (**A**) Culture the desired cancer cell lines at the specific cell numbers, (**B**) dilute them to the desired inoculum concentration, (**C**) inject desired cell numbers to create in vivo model, (**D**) when the tumor has reached the desired volume, the oncolytic virus is injected to inhibit its growth. (**E**) If the oncolytic virus is effective, tumor mass reduction will be observed. (**F**) Tumor growth will be minimal, and the subject will regain health.

**Figure 3:**
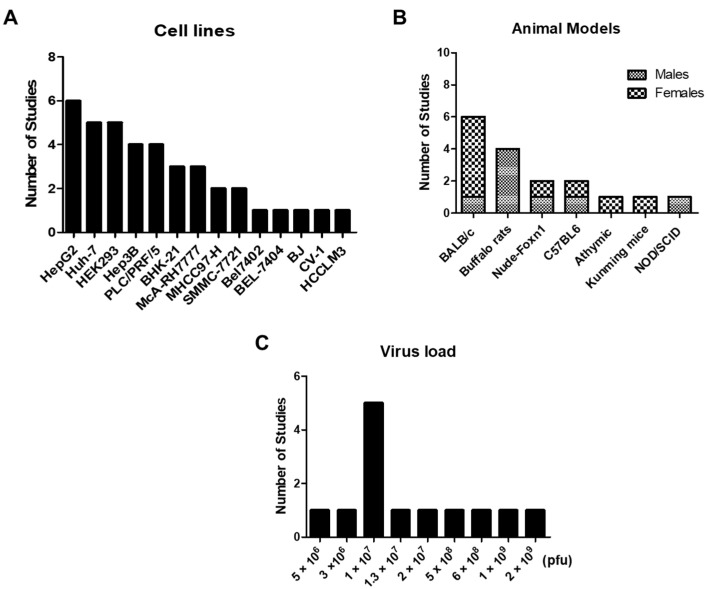
Distribution of studies according to the used (**A**) cell lines, (**B**) animal models, and (**C**) dose (the virus load) of in vivo inoculation.

We summarized in Figure 2 and Table 1 the experimental details of the oncolytic virus studies reviewed here. The most used cancer cell line was HepG2 (Figure 3A) and a number of implanted cancer cells used to establish in vivo tumor models (Figure 3A) varied in the 10^5^–10^7^ range. The major animal models (Figure 3B) were Balb/c nude mice (used in five studies) and buffalo rats (three studies), but there were also five other mouse models used, one in each of a handful of studies. Among the 13 studies, four each were focused on oncolytic vaccinia virus (VV), oncolytic adenovirus (AdV), and oncolytic vesicular stomatitis virus (VSV). Our search could identify one study each for Newcastle disease virus (NDV) and oncolytic avian reovirus (ARV). The dosage used for virus inoculation (Figure 3C) varied in the 10^6^–10^9^ range.

### 3.3. In Vivo Oncolytic Virotherapy

In this review, we aim to systematically analyze experimental approaches used in OVT and determine the status of the field. The survey of studies revealed the following five categories of OVs: VV, AdV, VSV, NDV, and ARV, that have been used with HCC models. We next discuss the body of work associated with each virus category under separate subheadings.

#### 3.3.1. Oncolytic Vaccinia Virus (VV)

VV is a member of the orthopoxvirus genus within the Chordopoxvirinae subfamily. VV has a linear, double-stranded, approximately 190 kb DNA genome that encodes approximately 200 genes. The virus particle has the form of a brick and measures 270 × 350 nm on average. In mammalian cells, the entire VV life cycle occurs within the cytoplasm [14]. Virion fusion with the membrane of the host cell allows for cell entry. VV includes an outer envelope and an internal membrane, as well as enzymes necessary for the initiation of viral transcription following infection. There are the following three stages of viral transcription: early, intermediate, and late, with each stage involving its own promoters and transcription factors. The benefits of VV may include, but are not limited to, the following: (1) its efficient life cycle, which produces mature virus progeny in only 6 h; (2) its three viral spread mechanisms, which ensure rapid and efficient virus dissemination; (3) its large viral genome, which allows it to accept up to 40 kilobases of foreign DNA; (4) its inability to cause disease in healthy humans; (5) due to its use as a smallpox vaccine, extensive clinical experience and knowledge of the virus are available. On the other hand, the viral genome encodes approximately 200 viral genes, 50 percent of which have unknown functions, which gives this virus an element of unpredictability [15].

The MHCC97-H xenograft mouse model was developed by subcutaneous (SC) injection of 4 × 10^6^ MHCC97-H cells into the right flank to investigate the anti-cancer effects of the combination treatment in vivo [16]. Luteolin inhibits the growth of cell lines derived from various types of tumors, and this effect is mediated by apoptosis promotion and proliferation inhibition. The mitochondrial translocation of Bax/Bak and the activation of JNK signaling cause luteolin-induced apoptosis in liver cancer cells. Thus, combining VV-IL 24 and luteolin would result in a more significant anti-cancer effect. Using a phosphate buffer saline (PBS) vehicle for intratumor (IT) injection of VV-IL24 and/or intraperitoneal injection of luteolin, viruses were administered to mice in the following four groups: (i) luteolin (50 mg/kg) alone; (ii) VV-IL24 2 × 10^7^ plaque forming unit (PFU) alone; (iii) VV-IL-24 2 × 10^7^ PFU together with luteolin (50 mg/kg); control injected with the vehicle alone. Tumor length and width were measured every five days, and tumor volume (mm^3^) was calculated using the (1/2) × length × width^2^ formula based on the approximation of tumor shape with an elongated ellipsoid. By therapy day 15, tumor growth curves clearly revealed that xenograft MHCC97-H tumor development was inhibited in the VV-IL-24 plus luteolin combination treatment group relative to the single treatment group. On treatment day 35, the tumor volume in the PBS, luteolin alone, and VV-IL-24 groups reached 3503 mm^3^, 3080 mm^3^, and 1088 mm^3^, respectively. In striking contrast, the tumor volume in the VV-IL-24 and luteolin combination group was a mere 105 mm^3^, indicating a remarkable restriction of tumor growth. Thus, the combination treatment exhibited a significantly more substantial anti-cancer impact in vivo than either VV-IL-24 or luteolin alone [16].

**Table 1:** Summary of the experimental details of the oncolytic virus studies reviewed here.

OV	Publication Author/Year	Animal, Sex ^1^, Age ^2^	Cell Line	Number of Cancer Cells ^3^, Route *	Tumor Measurement Frequency (Days)	Tumor Size at the Time of Virotherapy (mm^3^)	Agent/Virus ^4^	Virus Load ^5^ (PFU), Route *	Outcome (+/−) ^6^
Oncolytic vaccinia virus	Wang et al. (2021) [16]	BALB/c nude mice F, 5	MHCC97-H	4 × 10^6^ SC	5	80–120	Luteolin/VV-IL24	2 × 10^7^ IP, IT	VV-IL24/Luteolin + (*p* < 0.001)
	Zhang et al. (2019) [17]	C57BL6 mice F, 6	Bel7402	2.5 × 10^6^ SC	-	-	VV-IL-37	1 × 10^7^ IT	VV-IL-37 + (*p* < 0.01)
	Li et al. (2018) [18]	BALB/c nude mice F, 4–5	MHCC97-H,	2.5 × 10^6^ SC	5	120	OncoVV-TTL	1 × 10^7,^ IT	OncoVV-TTL+
	Gentschev et al. (2011) [19]	Nude-Foxn1 M/F, 6–8	Huh-7, PLC	5 × 10^6^ SC	3.5	-	GLV-1h68	5 × 10^6^ IV	GLV-1h68 PLC+, HuH7 -
Oncolytic adenovirus	Xie et al. (2018) [20]	BALB/c nude mice F, 4	Huh-7	3 × 10^6^ ^-^	-	80–100	Ad-sp-VGLL4	5 × 10^8^ ^-^	Ad-sp-VGLL4 + (*p* < 0.01)
	Zhang et al. (2016) [21]	NOD/SCID mice M, 4 BALB/C nude mice F, 5	PLC/PRF/5	2 × 10^6^ SC	3	100	GD55 ZD55	6 × 10^8^ IT	GD55 +
	Chen et al. (2011) [22]	BALB/c nude mice; M/F, 4–6	Hep3B	5 × 10^6^ SC	-	100–150	Ad5/35EGFP	1 × 10^9^ IT	SG600-p53- and SG635-p53 + (*p* < 0.001)
	Cao et al. (2011) [23]	athymic nude mice F, 4	Huh-7	5 × 10^6^ SC	7	90–120	SOCS3, IL-24/OAV	2 × 10^9^ IT	SOCS3, IL-24/OAV+ (*p* < 0.001)
Newcastle disease virus	Meng et al. (2020) [24]	C57BL6 mice M, 6	H22, Hepa 1–6	2 × 10^6^, 5 × 10^6^ IP, SC	-	-	Dichloroacetate/NDV	1 × 10^7^ IT	DCA/NDV +
Oncolytic avian reovirus	Cai et al. (2019) [25]	SPF Kunming mice F, 5	-	-	-	-	ARV S1133	3 ×10^6^ O, IM	ARV S1133+, No pathologic damage Apoptosis in cell line HepG2
Oncolytic vesicular stomatitis virus	Altomonte et al. (2008) [26]	Buffalo rats (IC) M, 6	-	-	-	-	rVSV-gG	1.3 × 10^7^ IV	rVSV-gG +, No pathologic damage to organs,
	Altomonte et al. (2009) [27]	Buffalo rats M, 5–7	-	-	-	-	rVSVUL141, rVSV-F	1 × 10^7^ IV	rVSV-F-, rVSV-UL141 + (*p* < 0.001)
	Altomonte et al. (2013) [28]	Buffalo rats M, 6	-	-	-	-	rVSV-LacZ, rVSV (M51R)	1 × 10^7^ IV	rVSV-LacZ + (*p* < 0.005)

**^1^** Animal sex, F—female; M—male. **^2^** Animal age, in weeks. **^3^** Number of cancer cells implanted in animal to establish in vivo tumor model. **^4^** Names of the viruses and genetic transfection agents are defined in the text. **^5^** Virus load used for in vivo inoculation. **^6^** Outcomes of experimental oncolytic viral therapy: **+** positive outcome oncolytic virus successfully suppressed the tumor without harming healthy cells; **−** negative outcome oncolytic virus failed to suppress the tumor or harmful to healthy cells. * Route of administration of cancer cells and oncolytic virus; IM—intramuscular, O—orally, SC—subcutaneous, IT—intratumor, IP—intraperitoneal, IV—intravenous.

Furthermore, the study showed that VV-IL-24 and luteolin worked together to limit the growth of liver cancer tumor xenografts as follows: On the seventh day after the last treatment, tumor tissue samples were taken from a mouse selected at random from each group. Examination using immunohistochemical (IHC) staining for the IL-24 protein revealed that IL-24 was expressed significantly higher in tumor tissue from the VV-IL-24 plus luteolin combination treatment group than in the other groups. CD31 is a vascular endothelial marker that has been linked to tumor growth, angiogenesis, and metastasis. Ki67 is a proliferative cell-associated nuclear antigen found in several cancers. Immunohistochemistry examination demonstrated that the CD31 and Ki67 staining in the combination treatment group was substantially less than in the VV-IL-24 or luteolin treatment groups. IHC labeling was also used to detect the expression of cleaved caspase 3. The results confirmed that the protein level of cleaved caspase 3 increased significantly more in the VV-IL-24 and luteolin combination treatment group than in the groups with either VV-IL-24 or luteolin alone. This result indicated that induction of cleaved caspase 3 may have contributed to the enhancement of the in vivo anti-tumor efficacy of the VV-IL-24 plus luteolin combination therapy.

Additionally, these findings suggest that VV-IL-24 paired with luteolin had a higher anti-tumor efficiency in vivo, and the pairing may have contributed to increased IL-24 gene expression as well as the prevention of tumor cell proliferation and angiogenesis. In fact, HE-staining revealed that the combination treatment with VV-IL-24 and luteolin caused a more severe cytopathic impact in tumor tissues than either VV-IL-24 or luteolin treatment alone. Moreover, the liver, kidney, and spleen tissues from animals in the combination group had no or little cell damage, suggesting that VV-IL-24 and luteolin had no or little harmful effects on these tissues [16].

Another study developed an HCC model by subcutaneously implanting 2.5 × 10^6^ Bel7402 cells into female C57BL/6 mice [17]. Four weeks after implantation, the animals were injected intratumorally with 1 × 10^7^ PFU of either VV-IL-37-GFP or VV-mock-GFP. Three weeks later, the mice were sacrificed, and the volume and weight of the tumors were measured. The tumors were smaller and much lighter in the VV-IL-37-GFP group than in the VV-mock-GFP group. The results of a Western blot assay indicated that VV-IL-37-GFP infection increased IL-37 protein expression in HCC mice. The ratio of p-STAT3/STAT3 protein expression in tumor tissue was considerably lower in the VV-IL-37-GFP group than in the VV-mock-GFP group. In addition, VV-IL-37-GFP infection significantly increased the levels of IL2, IFN, and TNF in HCC mice. These findings were consistent with the antiproliferation impact of VV-IL-37-GFP infection reported by in vitro studies and suggested that VV-IL-37-GFP induced a potent anti-tumor response in HCC in vivo [15].

#### 3.3.2. Oncolytic Adenovirus (Adv)

Adv is one of the most frequently used OVs due to its ability to lyse tumor cells and stimulate the immune system. Adv is a non-enveloped, double-stranded, linear DNA virus with an icosahedral capsid composed primarily of hexon, penton, and fiber proteins. The genome is approximately 36 kb long and can encode over 40 gene products [29]. Based on their transcription start time, these gene products are divided into the following three subtypes: early, middle, and late stages. Early gene products are primarily responsible for gene regulation, which includes the E region, whereas late gene products are primarily responsible for coding structural proteins, which includes the L region [30]. Adv replicates continuously in tumor cells, eventually lysing tumor cells and infecting other tumor cells through the same mechanism of action. Therapeutic genes are frequently inserted into the vector due to its high loading capacity. Due to the continuous replication and accumulation of adenoviruses in tumor cells, therapeutic genes are expressed and therefore spread, playing a synergistic role in antitumor activity [31].

The BALB/c nude mice were injected with 3 × 10^6^ HuH7 cells to produce the HCC xenograft model [20]. When the tumor volume reached 80–100 mm^3^, the animals were randomly separated into three groups (eight mice per group) and injected daily with 5 × 10^8^ Adv or PBS (for a total of 4 times). The tumor was measured twice a week and the volume was estimated using the elongated ellipsoid formula (1/2 length × width^2^). A comparison of the tumor volume growth curves of the PBS and Ad-sp groups revealed that Ad-sp-VGLL4 inhibits the growth of tumors. Terminal deoxynucleotidyl transferase dUTP Nick-End Labeling (TUNEL) studies of HCC tumor tissue sections confirmed that Ad-sp-VGLL4 promoted apoptosis in HCC in vivo, and HE-staining demonstrated that Ad-sp-VGLL4 inhibited angiogenesis and caused damage in HCC. Together, these results suggest that Ad-sp-VGLL4 has therapeutic potential for HCC [20].

To assess the anti-tumor effectiveness of the adenovirus GD55 and compare it to the common oncolytic virus ZD55 as a control, subcutaneous xenograft models in nude mice were created by injecting the animals with PLC/PRF/5 sphere cells [21]. The results of tumor growth showed that GD55-infected xenograft tumors grew at a much slower rate compared to those in the PBS or ZD55 groups. Moreover, HE-staining revealed that GD55 induced more severe cell death and necrosis in the tumor mass than ZD55. Importantly, GD55, similarly to the PBS-treated group, did not discernibly harm the liver tissue. However, compared to ZD55, GD55 produced a more pronounced death of tumor cells, as determined by the TUNEL assay, and decreased cell proliferation and angiogenesis in xenograft tumors to a larger extent, as measured by IHC for Ki67 and CD31. These in vivo results indicate that GD55 might be considerably more effective in limiting the growth of tumors formed from PLC/PRF/5 sphere cells than the common oncolytic virus ZD55 [21].

Each mouse was injected subcutaneously on the right flank with 5 × 10^6^ Hep3B human HCC cells suspended in 100 μL of PBS [22]. When tumors reached 100–150 mm^3^ in volume, 40 mice were randomly assigned to one of the following five therapy groups (*n* = 8 per group): SG600, SG635, SG600-p53, SG635-p53, or PBS. Five intratumoral (IT) injections of 2 × 10^8^ PFU viruses diluted in 100 μL of PBS were administered to the mice in the adenovirus treatment groups every other day for a total of 1 × 10^9^ PFU. The control group was injected with the same volume of PBS alone. Using a caliper, tumor development was measured on days 0, 5, 10, 14, 21, 28, and 35 following the last injection. The elongated ellipsoid formula (given above) was used to compute tumor volume (V). Compared to the PBS-treated control group, all four virus-treated groups had measurable anti-cancer activity 21 days after the last treatment. Compared to the control group, the tumor volume decrease rates in the SG635-p53-treated group were 92%, 99%, and 97% on days 14, 21, and 28 after treatment, respectively. For the SG600-p53-treated group, the corresponding measures, at 44%, 65%, and 77%, respectively, were weaker. Moreover, over a 35-day post-injection observation period, the mean tumor volume of mice treated with SG600-p53 increased 6.7-fold, to 1212 mm^3^, but the tumor volume in mice treated with SG635-p53 increased only 1.7-fold, to 322 mm^3^ (*p* < 0.001) [22].

By post-treatment day 70, all animals in the PBS, SG600, and SG635 groups were euthanized due to massive tumors, with median survival periods of 28, 38, and 44 days, respectively. Thus, compared to the control group, the SG600-p53 and SG635-p53-treated animals benefited from significantly extended survival (*p* < 0.001). Four of six mice treated with SG600-p53 died by day 70 post-treatment, and the median survival duration was 61 days. In contrast, all six mice in the SG635-p53-treated group were still alive at the conclusion of the experiment, and the median survival time had not been attained.

To confirm that the improved therapeutic efficacy was a result of enhanced viral internalization and multiplication, two mice from each group were sacrificed five days following the last injection, and their tumors were collected for histological analysis. Sections of subcutaneous tumors stained with HE revealed numerous significant necrotic patches in the SG635-p53-treated tumor tissue. In contrast, in the control and SG600-p53-treated groups, localized necrosis patches were tiny or fully absent. An immunohistochemistry analysis revealed that expression levels of the E1A protein in tumor tissues for the SG635-p53-treated group were greater than for the SG600-p53-treated group. In addition, the majority of cancer cells around necrotic areas were positive for the p53 protein in tumor tissues from the SG635-p53-treated group. In contrast, in the control and SG600-p53-treated groups, p53-positive cells were few or absent. Thus, according to these findings, SG635-p53 replicated and disseminated more rapidly than SG600-p53 in liver cancer tissues in vivo [22].

#### 3.3.3. Oncolytic Vesicular Stomatitis (VSV)

VSV, a member of the Rhabdoviridae family, is an encapsulated, negative-strand RNA virus with a broad range of host species. VSV replicates preferentially within tumor cells due to deficiencies in antiviral type I interferon responses, has a short replication cycle, and an ability to attain high titers in the majority of rat and human tumor cells. These features make VSV a promising oncolytic agent. We identified three recent studies utilizing VSV for the oncolytic treatment of HCC. Altomonte et al. (2008) [26] described the molecular synthesis and characterization of a new rVSV vector that encodes the secreted version of the equine herpesvirus-1 glycoprotein G, which is a viral chemokine binding protein (vCKBP) with high affinity for C, CC, and CXC chemokines. Immunocompetent rats with syngeneic and multifocal HCC lesions in the livers were found to have elevated tumor necrosis and significantly prolonged survival when the rVSV vector inhibited natural killer cell (NK) migration to the tumor sites. This resulted in greatly enhanced intratumoral virus replication, which led to increased tumor necrosis and substantially prolonged survival.

Additionally, Altomonte et al. (2009) [27] introduced rVSV-UL141, a recombinant virus that contained a protein from the human cytomegalovirus known to downregulate CD155, the NK cell-activating ligand, to counteract these cells’ antiviral activity. The modified vector inhibited the recruitment of NK cells in vitro and lowered the intra-tumoral accumulation of NK and NKT cells in vivo. In immunocompetent buffalo rats with orthotopic, multifocal HCC lesions, hepatic artery infusion of rVSV-UL141 resulted in an (one-log unit) increased intratumoral viral replication over the replication levels of a control rVSV vector, leading to an accelerated tumor necrosis and significant extension of life in the treated animals. Importantly, these outcomes were obtained in the absence of any obvious toxicities. These results suggest a promising potential for this technique to produce effective and safe oncolytic medicines to treat multifocal HCC and perhaps a variety of other malignancies in the future.

Similarly, using a thioacetamide-induced rat model of fibrosis, Altomonte et al. (2013) [28] demonstrated that VSV, administered via hepatic arterial infusion, not only retained its ability to kill tumor cells efficiently but also possessed antifibrotic properties that resulted in the unique benefit of concurrently reversing fibrotic progression. In these in vivo experiments, the livers of rats with thioacetamide-induced fibrosis were treated with either PBS or 10^7^ PFU of the rVSV-LacZ vector, and paraffin sections were prepared 24 h after therapy. The antifibrotic effects of VSV, revealed by an examination using αSMA-specific immunohistochemical staining, may be the consequence of the following three independent but perhaps interrelated mechanisms: (i) direct cell death of infected HSCs, (ii) recruitment of activated NK cells, and (iii) gene expression regulation in favor of the fibrotic resolution.

#### 3.3.4. Newcastle Disease Virus (NDV)

NDV belongs to the genus avulavirus in the Paramyxoviridae family. Six structural genes are encoded in its tiny genome (15.2 kb, negative single-stranded RNA). In the study, using the ascitic HCC model and subcutaneous HCC mode, tumors were injected intraperitoneally (IP) and SC on day 0 using 2 × 10^6^ H22 and 5 × 10^6^ Hepa 1–6 cancer cells, respectively [24]. On day 2, H22-bearing mice were randomly assigned to one of four treatment groups (untreated, dichloroacetate (DCA), NDV, and NDV-DCA). Every day from day 3 to day 17, 200 mg/kg DCA or an equivalent amount of sterile water was injected intragastrically (IE) into mice. On days 4, 5, 8, 9, 12, and 13, mice were injected intraperitoneally (IP) with 1 × 10^7^ PFU of NDV or an equivalent amount of PBS. On days 7, 10, and 13, mice were injected intratumorally with 1 × 10^7^ PFU per mouse of NDV, respectively. Meanwhile, on day 5, mice carrying Hepa 1–6 were randomly assigned to four treatment groups (untreated, DCA, NDV, and NDV-DCA). On days 7, 10, and 13, mice were injected IT with 1 × 10^7^ pfu per mouse of NDV, respectively. Every day from day 7 to day 20, mice were administered 200 mg/kg DCA or an equivalent volume of sterile water.

On days 10 and 15, a 500 μL sample of ascites was collected and used to measure cell quantity, NDV replication, antiviral gene expression, immune cell infiltration, IFN-ELISpot, IDO1 expression, and STAT-phosphorylation. Every two to three days, the tumor volume was measured with a caliper and computed using the usual elongated ellipsoid formula. The body weight was measured every other day, and the survival rate was measured daily. Mice were euthanized by cervical dislocation when the volume of the tumor reached 3 cm^3^ or when the mice seemed lifeless.

The combination of NDV and DCA reduced ascitic cells in the mouse ascitic HCC model, which prolonged survival (25% of combination-treated mice survived for more than 60 days). In addition, DCA improved the therapeutic efficacy of NDV and extended mouse survival in a subcutaneous HCC model. Two of seven mice responded completely to the combination treatment. In either the ascitic or subcutaneous HCC models, no obvious therapy-related side effects or bodyweight loss were observed. These findings suggest that DCA improves NDV’s antitumor efficacy in HCC [24].

#### 3.3.5. Oncolytic Avian Reovirus (ARV)

ARVs are nonenveloped viruses that replicate in the cytoplasm of infected cells. They are members of the Reoviridae family and have 10 double-stranded RNA genome segments. ARV is an oncolytic virus that has been the focus of anticancer research.

For this study, five-week-old female SPF Kunming mice (*n* = 60) were used as in vivo hosts to investigate the oncolytic benefit of treatments with avian reoviruses [25]. Animals were assigned to the following four random groups: the oral group (*n* = 15), the intramuscular group (*n* = 15), and two control groups (*n* = 15, each). The oral and intramuscular groups were given 3106TCID50/0.2 mL of ARV S1133, respectively, while the control group was given 0.2 mL of PBS. Three mice were selected randomly on post-infection days 1, 3, 5, 7, and 14 to collect samples of their organs (heart, liver, spleen, lung, and kidney) for virus detection via qPCR and tissue pathology using HE-staining. In the meantime, the vitals, weight, and mortality were observed daily for 14 days. Infected Kunming mice showed no clinical signs, such as fever, dyspnea, anorexia, weight loss, behavioral irregularities, or mortality. In addition, no difference was observed between the oral and intramuscular groups. In the oral group, the viral load peak point appeared on post-infection day 3 in all tissues except spleen tissue, where it occurred on post-infection day 5 [25].

## 4. Discussion

In this review, we discuss recent in vivo work on OV in murine HCC models. An exhaustive and rigorous search of four major databases (PubMed, Cochrane, Embase, and Web of Science) identified a total of 13 novel studies using OVT in HCC. We categorized the studies by the groups of viruses used and discussed the mode of viral action the studies revealed. Our review highlights a body of evidence that suggests that OVT could be a preferred future technology for cancer treatment. However, for this to be realized, OVT efficacy needs to be further improved, which will require future investigations to address the following three areas of current technological shortcomings: (i) optimization/enhancement of systemic viral delivery, (ii) intertumoral virus dispersion, and (iii) anti-cancer immunity cross-priming.

These shortcomings can be optimized by choosing a host with a TME close to the humans. To address that, many in vivo models are available in the field, each with their own advantages and disadvantages. Immune competent models considered usually include chemically induced models, syngeneic transplanted models, and genetically engineered models [32]. A key barrier to the engraftment of human cancer cells in immunocompetent rodents is robust xenogeneic immune rejection [33]. The xenograft model can provide better insights to address these issues; therefore, in our studies, the majorly used model was the xenograft cell line-based model where the cells were inoculated inside the immune-deficient mice and the effect of OV on the tumor was recorded via injecting the OV intratumorally once the tumor reached the specific size. These transplantation models allow rapid evaluation of potential OVT and can be a potential first-line checkpoint. However, because cancer cell lines contain multiple mutations from the start and acquire additional aberrations when cultured in vitro for extended periods of time, these inoculation models might not exactly match the morphology and genetic heterogeneity of human cancers and are often poor predictors of clinical response [13].

OVT can be further explored or improved by using GEMMs or humanized mice [34]. Despite their high cost, they are closer to mimicking the TME of human tumors than their available counterparts. Humanized mice may be the first choice among these, as engrafting human peripheral blood mononuclear cells (hPBMCs) into severely immunodeficient mice is a simple and cost-effective method for producing humanized mice. hPBMC engraftment allows human tumor xenografts from cell lines or tumor explants to be studied in an autologous or heterologous immunologic context, but it has the drawback of causing a robust human xenograft versus host disease (xGvHD) a few weeks after hPBMC engraftment. The xGvHD is thought to be caused by a mismatch in the major histocompatibility complex (MHC) between human T cells and mouse cells [12]. Meanwhile, GEMMs of de novo tumorigenesis are the systems of choice for in vivo analysis of the cell-intrinsic and cell-extrinsic processes that contribute to cancer initiation, progression, and metastasis; however, one major limitation of GEMMs is that developing and validating these models is time-consuming, laborious, and costly [13].

In recent years, OVT, a novel type of anti-cancer therapy, has attracted a growing amount of public attention due to the ability of the method to destroy tumor cells without harming normal cells. Even though it is a promising cancer treatment option, OVT research still has many obstacles to overcome in the three areas listed above before OVT becomes the treatment of choice in clinical practice. A further, even greater challenge will likely be the harmonization of solutions to each of these problems; nevertheless, this goal is unquestionably attainable [35]. For instance, suppressing the immune system may increase intertumoral spread while diminishing the anti-cancer immune response’s cross-priming. In contrast, boosting immunity may improve cross-priming but at the expense of limiting intertumoral virus spread, thus acting against the foundation of oncolytic tumor debulking. Numerous methods, asserted as ‘solutions’, have been developed to date, but they have only been analyzed in artificial model systems that lack the capability to reveal the positive and negative consequences of a given modification to all aspects of the overall treatment paradigm. This, in turn, highlights yet another significant challenge for the field, which is the urgent development of more accurate model systems that can reliably capture the complexity of human OVT scenarios.

Furthermore, replication competent oncolytic VVs that selectively infect tumors are emerging as promising therapeutic targets for liver cancer [4]. VV has multiple anti-tumor mechanisms, including direct oncolysis, suppression of the immune response induced by tumors, and anti-angiogenesis [36,37]. As a promising example, the combination of VV-IL-24 and luteolin was significantly more effective as a suppressor of tumor growth in the MHCC97-H nude mouse xenograft model than either luteolin or VV-IL-24 alone. Both luteolin and IL-24 have been shown to inhibit tumor growth through the activation of the JNK signaling pathway, but whether the combination therapy can enhance the activation of the JNK signaling pathway still needs further studies. IHC revealed lowered expression levels for CD31 and Ki67 after the combination treatment than after the single-agent treatments, suggesting that the combination therapy inhibits the proliferation of liver cancer cells and angiogenesis in liver tumors.

Moreover, the combination of VV-IL-24 and luteolin produced more extensive cell death in the tumor tissue than treatments using each component alone, with no or little toxic effects on the liver, kidney, and spleen tissues. Recent studies have shown that MDA-7/IL-24 controls several microRNAs, such as miR-221, that are upregulated in diverse types of cancer [38]. MDA-7/IL-24 downregulated miR-221, which in turn induced Beclin-1, leading to autophagy. IL-24 has been shown to promote the populations of CD4+ and CD8+ T cells in diverse cancer models [39,40]. The ERK signaling pathway is necessary for the replication of the VV [41].

The combination of gene therapy with virotherapy for cancer treatment has gathered significant interest and become a trend in cancer biotherapy. A technique known as ‘Cancer Targeting Gene-Viro-Therapy’ (CTGVT) or ‘Gene Armed Oncolytic Viral Therapy’ has been developed in which an anti-tumor gene is inserted into an oncolytic viral vector [23]. Because of its tumor selectivity, safety, and efficacy, Adv is regarded as one of the most promising OVs and is intensively researched. In addition to its remarkable ability to be combined with other therapies, such as chemotherapy and immunotherapy, Adv has the remarkable ability to maintain foreign gene expression in live cells [42,43,44]. Numerous experiments to date have demonstrated that Adv carrying anti-cancer genes exhibit remarkable anti-tumor activity in vivo and in vitro [20,21,22,23,45].

Most Adv used in cancer therapy are based on adenovirus serotype 5 (Ad5), which is a member of subgroup C and requires a coxsackie–adenovirus receptor (CAR) on target cells for successful transduction [46,47]. It is known that CAR expression is reduced or lost during the malignant progression of certain tumors, including HCC [48,49,50]. These constraints impede the transduction of Ad5-based vectors into certain types of tumor cells, resulting in diminished anti-tumor efficacy. In contrast, CD46, the cellular receptor for Ad35, is markedly upregulated in numerous types of malignant tumor cells, such as HCC cells [46,51]. To circumvent the restriction of CAR-dependent cell entry, researchers have recently developed new fiber chimeric adenoviral vectors. They exchanged the knob and shaft of the Ad5 fiber, such as the Ad35 fiber, which can recognize the abundant CD46 receptor on tumor cells or the vestigial-like family (VGLL), which is known to restrain tumor growth through suppressing cell proliferation [20,52].

In vivo experiments demonstrated that GP73-regulated GD55 OAV inhibited, to a certain extent, tumor growth in BALB/c nude mice xenografted with liver sphere cells via apoptosis induction, anti-proliferative, and anti-angiogenesis mechanisms within implanted tumors. These observations are consistent with some studies involving other cancer-targeting Adv types [53]. The hope is that, through further engineered modification, the ability of GD55 to target liver cancer stem cells (CSCs) will be improved. Based on the molecular differences between CSCs and non-CSCs, it may serve as a carrier for liver CSC-specific inhibition genes or RNAi designed to target the key transcription factors (such as Nanog) and the central signaling nodes (such as AKT/PI3K). The introduction of immune-promoting genes such as GM-CSF can stimulate immune responses against tumors, and the delivery of TRAIL or IL-24 effectively eliminates cancer cells via apoptosis, playing crucial killing roles not only in CSCs but also in non-CSCs [5,54].

Ras mutations can promote reovirus oncolysis. The Ras pathway is essential for sensitizing cancer cells to reovirus. Ras accumulation has been reported to induce apoptosis in Ras-transformed fibroblasts. Additionally, reovirus significantly induces apoptotic cell death in the gastrointestinal stromal tumor cells via the Fas-FasL pathway. Using histopathologic examinations, the current study determined that ARV did not cause pathogenicity in experimental groups or control groups, confirming the safety of ARV and suggesting the tissue tropism of ARV based on the results of dynamic tissue distribution of ARV S1133. Importantly, the trend of tissue tropism of the five organs from all infected mice in the study according to the foldchange (three highest) was the following: liver, kidney, and spleen, whether in oral groups or in intramuscular injection groups, and the peak foldchange of liver tissue was exceptionally high, so it can be assumed that the ARV would be most effective against liver cancers, followed by kidney and spleen cancers [25].

Several clinical and preclinical experiments have demonstrated that NDV is effective at treating cancers without negatively impacting normal cells [55,56]. In addition to its direct oncolytic effect, there is mounting evidence that NDV affects the immune system, causing tumor rejection via a specific or generic anti-tumor immune response [57,58]. Its interactions with antiviral type I interferons (IFNs) and the apoptotic pathway determine tumor cell selectivity [59,60]. Localized therapy with oncolytic NDV induces inflammatory immune cell infiltration of NK cells 1.1+, CD3+CD8+, CD11b+ lymphocytes, and monocytes) in both injected and distant tumors without distant virus spread, rendering the tumors susceptible to systemic immunotherapy [61]. The NDV therapy depends on CD8+ T cells and NK cells, and type I IFNs, but not CD4+ lymphocytes [61,62].

Furthermore, aerobic glycolysis is prevalent in most malignant cells (Warburg effect) and is crucial to the formation of an immunosuppressive TME [63]. Notably, glycolysis inside tumor cells has been demonstrated to deplete extracellular glucose, hence limiting the access of T cells to glucose. This decreased glucose availability inhibits glycolytic metabolism in T cells, which is linked with diminished proliferation and effector function [64]. In addition, the buildup of lactate in the TME because of glycolysis has a severe effect on the functional characteristics of T cells and NK cells [65,66]. It has been observed that inhibiting glycolysis increases the anti-tumor T-cell response in the TME [67,68]. Coincidentally, viral infection, such as measles virus, adenovirus, human immunodeficiency virus, or human cytomegalovirus, substantially alters host cellular glucose metabolism to high-level glycolysis, which might be an impediment to oncolytic virus-immunotherapy [69,70,71,72].

Thus, one way to increase the effect of virotherapy is by combining it with some immune modulator, such as DCA, which has a greater influence on lactate generation than on glucose consumption, consistent with an increase in oxidative phosphorylation compared to glycolysis [24]. Multiple studies have shown that various OVs, including adenovirus and measles virus, may increase aerobic glycolysis and lactate production. Considering the metabolic rivalry between cancer cells and immune cells in the TME, the restriction of glucose consumption by DCA may increase glucose availability to immune cells, thereby promoting the proliferation and effector function of immune cells [69]. In such cases, DCA therapy may enhance the capacity of OVs to enhance the anti-tumor immune response. To this point, DCA therapy was shown to boost the anti-tumor activity of immune cells in the TME by decreasing NDV-induced lactate generation in HCC [24]. Therefore, the combination of DCA and OV may prove to be an effective immune modulator in OVT.

In addition, in the absence of effective antifibrotic therapy, patients with established cirrhosis have a high risk of developing HCC, which adds a significant further challenge to the treatment of HCC. Therefore, it is essential that novel therapies are developed to treat hepatic fibrosis in a safe and effective manner. One possible option is VSV, which replicates selectively in tumor cells and activates hepatic stellate cells (HSCs), making it an ideal agent for treating HCC and the underlying fibrosis simultaneously. If VSV is administered during the early stages of fibrosis development, the progression of the disease may be halted, and the onset of HCC and other complications of end-stage liver disease may be delayed or even prevented [28].

While we did our best to acquire and search all possible databases for this review, we were forced to exclude studies not published in the English language because of the technical difficulty of accurately extracting data, which could have adversely biased our review. Despite these limitations, we hope that our review can be a useful reference for investigators who seek to embark on designing an OVT study or experimental protocol.

## 5. Conclusions

In conclusion, murine models, despite their imperfect representation of in vivo OVT in humans, can help us improve OVT for more effective clinical trials. Further research needs to address improvement in the following three areas: (i) systemic viral delivery optimization/enhancement, (ii) intertumoral virus dispersion, and (iii) anti-cancer immunity cross-priming. With improvements in those areas, OV has the potential to gain increased efficacy and become a preferred cancer therapy method in the future.

## Data Availability

All data needed to support the conclusions are present in the paper. Additional data related to this paper may be requested from the authors.
